# Patient safety and safety culture in primary health care: a systematic review

**DOI:** 10.1186/s12875-018-0793-7

**Published:** 2018-06-30

**Authors:** Muna Habib AL. Lawati, Sarah Dennis, Stephanie D. Short, Nadia Noor Abdulhadi

**Affiliations:** 1 Faculty of Health Sciences, Discipline of Behavioral and Social Sciences in Health, The University of Sydney, Science Road, Sydney, NSW 2006 Australia; 2 Department of Quality Assurance and Patient Safety, Ministry of Health, P.O.Box, 626, Wadi Al Kabir, 117 Muscat, PC Oman; 3 Ingham Institute for Applied Medical Research, Campbell Street, Liverpool, NSW 2170 Australia; 4 Faculty of Health Sciences, Discipline of Physiotherapy, The University of Sydney, 71 East Street, Lidcombe, NSW 2141 Australia; 5 Directorate General of Planning and Study, Ministry of Health, Muscat, Oman

**Keywords:** Patent safety, Safety culture, Primary care, Gulf countries, Oman

## Abstract

**Background:**

Patient safety in primary care is an emerging field of research with a growing evidence base in western countries but little has been explored in the Gulf Cooperation Council Countries (GCC) including the Sultanate of Oman. This study aimed to review the literature on the safety culture and patient safety measures used globally to inform the development of safety culture among health care workers in primary care with a particular focus on the Middle East.

**Methods:**

A systematic review of the literature. Searches were undertaken using Medline, EMBASE, CINAHL and Scopus from the year 2000 to 2014. Terms defining safety culture were combined with terms identifying patient safety and primary care.

**Results:**

The database searches identified 3072 papers that were screened for inclusion in the review. After the screening and verification, data were extracted from 28 papers that described safety culture in primary care. The global distribution of the articles is as follows: the Netherlands (7), the United States (5), Germany (4), the United Kingdom (1), Australia, Canada and Brazil (two for each country), and with one each from Turkey, Iran, Saudi Arabia and Kuwait. The characteristics of the included studies were grouped under the following themes: safety culture in primary care, incident reporting, safety climate and adverse events. The most common theme from 2011 onwards was the assessment of safety culture in primary care (13 studies, 46%). The most commonly used safety culture assessment tool is the Hospital survey on patient safety culture (HSOPSC) which has been used in developing countries in the Middle East.

**Conclusions:**

This systematic review reveals that the most important first step is the assessment of safety culture in primary care which will provide a basic understanding to safety-related perceptions of health care providers. The HSOPSC has been commonly used in Kuwait, Turkey, and Iran.

**Electronic supplementary material:**

The online version of this article (10.1186/s12875-018-0793-7) contains supplementary material, which is available to authorized users.

## Background

The World Health Organization (WHO) defines patient safety as “the prevention of errors and adverse effects to patients associated with health care” and “to do no harm to patients” [1, 2]. There are millions of patients globally who suffer disabilities, injuries or death each year due to unsafe medical practices [3]. This has led to the wider recognition of the importance of patient safety, the incorporation of patient safety approaches into the strategic plans of health care organizations and a growing body of research in this field [4]. “To Err is Human: Building a Safer Health System” was published in 1999 by the Institute of Medicine (IOM), it emphasized that safety was the key fundamental concern. This was a landmark publication for patient safety and warned of errors in health care and the potential for patient harm [5]. Patient safety in primary care has not been explored to the same extent as in the hospital settings [6] however more recently there has been more research emerging in primary care [7–10]. Achieving a culture of safety requires an understanding of the values, attitudes, beliefs and norms that are important to health care organization and what attitudes and behaviors are appropriate and expected for patient safety [10].

This systematic review aimed to identify the patient safety measures used globally to assess the effectiveness of safety culture in primary care. The outcome of this study will help to inform strategies for patient safety for primary care in Oman in order to accomplish the 2050 vision. The specific research questions for this review were:

1. What processes or systems are in place to facilitate a safety culture in in primary care?
2. What are the measures used globally to assess the effectiveness of safety culture in primary care?
3. What is the impact of safety culture in primary care?

## Methods

A systematic review of the published literature from 2000 to 2014 was conducted. This date range was chosen because it followed the publication of “To Err is Human” in 1999 [5]. The databases used to identify the articles were Medline, Embase, CINAHL and Scopus. The terms used in Medline search were *Health System, Safety Culture, Patient Safety, Primary Health care, Adverse Event, Health Care Professionals and Health Care Managers*.

There were several key definitions used to scope the review and inform the inclusion and exclusion criteria:

1. Patient Safety: WHO defines patient safety “as the absence of preventable harm to a patient during the process of health care” [1].
2. Safety Culture: Defined “as shared values, attitudes, perceptions, competencies and patterns of behaviors”.
3. Primary Care: WHO defined primary care “as socially appropriate, universally accessible, scientifically sound first level care provided by a suitably trained workforce supported by integrated referral systems (to secondary care or tertiary care) and in a way that gives priority to those most needed, maximizes community and individual self-reliance and participation and involves collaboration with other sectors. It includes the following: health promotion, illness prevention, care of the sick, advocacy and community development” [11].

Articles were included in the review if they were published in the year 2000 or later and met the following four inclusion criteria:

1. They reported on the use of patient safety tools or approaches or mechanisms or procedures used in primary health care with an impact on patient care (outcome) measured.
2. If they were contained any of the following methodologies; systematic review, intervention study (randomized controlled trials), descriptive study or qualitative design.
3. They discussed patient safety in primary care, or safety culture in primary care.
4. Published in English.

Articles were excluded if they were opinion papers/essays, editorial reviews, interviews, comments or narrative reviews.

After removal of the duplicates and papers with no abstracts, the titles and abstracts of 61 papers were screened by two researchers (MA and NN). The full text of all articles remaining were obtained and reviewed by two researchers (MA and NN). The full text articles were read and those that met the inclusion criteria were included in the review. The flow chart in Additional file 1 illustrates the selection process by using Preferred Reporting Items for Systematic Reviews and Meta-Analyses (PRISMA) flowchart [12].

The following information was extracted from the included articles: authors, year of publication, title and aims, objectives, methods, country and key findings. To assess the quality three different tools were used according to study design. Systematic reviews were evaluated by Assessing Methodological Quality of Systematic Review (AMSTAR), quantitative studies were assessed by Effective Public Health Practice Project (EPHPP) and cross sectional studies were evaluated by using Strengthening the Reporting of Observational studies in Epidemiology (STROBE) [13].

## Results

The database searches identified 3072 papers that were screened for inclusion in the review. After title and abstract screening there were 61 remaining papers that described interventions in safety culture in primary care. Following verification and data extraction there were a total number of 28 articles included in the systematic review (Additional file 1). The global distribution of the articles are as follows: the Netherlands (7), the United States (5), Germany (4), Australia, Canada and Brazil (two for each country), the United Kingdom (1), and with one each from Turkey, Iran, Saudi Arabia and Kuwait. The characteristics of the included studies grouped under the following themes: safety culture in primary care, incident reporting, safety climate and adverse events are specified in Table 1.

**Table 1:** Characteristics of the selected studies in the systematic review (studies categorized by themes)

Author and year	Title	Study design	Study Results and significant conclusions	Quality assessments
Safety Culture in primary care setting				
Kirk S [26] 2007	Patient safety culture in primary care; developing a theoretical framework for practical use.	Literature review followed by semi-structured interviews.	Study details development of the Manchester Patient Safety Framework	
Bodur S [8] 2009	A survey on patient safety in primary healthcare services in Turkey	Cross sectional study	Hospital survey on patient safety survey was adapted with modification to fit the Turkish primary care context. Positive responses were highest for teamwork within the units (76%) and lowest for events reporting (59%) and non-punitive response to errors (18%). Health center administrator must focus on improving patient safety culture and encourage staff to report errors without fear.	All items of STROBE statement covered
Dorien LM Zwart [22] 2011	Patient safety culture measurement in general practice. Clinimetric properties of ‘SCOPE’	Descriptive Cross sectional study	88.8% completed the questionnaire, out of which 25% were GPs, 60% medical administrative assistants and 15% nurses. SCOPE seems a suitable tool to measure safety culture in general practice	All items of STROBE statement covered
Nargis T [7] 2012	The first study of patient safety culture in the Iranian primary health care.	Cross sectional study	Teamwork across the units scored the highest 77.7%, continuous organization learning scored 72% and the lowest was non-punitive response to error 17%.	All items of STROBE statement covered
Jacobs L [27] 2012	Creating a culture of patient safety in primary care physicians group.	Proactive approach Case study	Study based on adaptation of medical risk management strategy to help create a culture of safety in primary care. This led to reduction of malpractice claims and enhanced learning experience among physicians.	All items of STROBE statement covered
Benjamin H [20] 2012	Better medical office safety culture is not associated with better scores on quality measures.	Cross section study	Response rate was 79%, significate variations on safety culture scores and quality scores. There was no association between safety culture and quality outcome measures.	All items of STROBE statement covered
Yahia M [21] 2013	Attitude of primary care physicians toward safety in Aseer region, Saudi Arabia	Cross sectional study	Highest score was given to reduction of medical errors (6.2 points). Followed by training and learning on patient safety (6 and 5.9). Undergraduate training was given the least score and participants did not agree that errors were due to nurses or doctor’s carelessness.	All items of STROBE statement covered
Lucine M [29] 2013	Is health professional’s perception of patient safety related to figures on safety incidents?	Retrospective Observational study	Communication breakdown inside or outside the practice are threats to patient safety. The study indicates that assessments of professional’s perception are complementary to observed safety incidents.	All items of STROBE statement covered
Fernando P [18] 2013	Patient safety culture in primary health care.	Cross sectional study	Working conditions, teamwork climate, communication and management of healthcare were significate with patient safety culture.	All items of STROBE statement covered
Maha G [10] 2014	Assessment of patient safety culture in primary health care setting in Kuwait.	Cross sectional studies	Hospital survey on patient safety survey was adapted with modification to fit the Kuwaiti primary care context. Dimensions with low positivity were: the non-punitive response to errors, frequency to error reporting, staffing, communication openness and center handoffs. High positivity was teamwork within the unit and organizational learning. Overall the safety culture is not strong in Kuwait.	All items of STROBE statement covered
Natasha J [24] 2014	Improving patient safety in primary care: a systematic review.	Systematic review	2 articles selected which provide basic understanding of improvement strategies in primary care, low level of evidence	9/11 using AMSTAR
Hoffmann B [25] 2014	Effects of a team based assessment and intervention on patient safety culture in general practice: an open randomized controlled trail.	Randomized control trail	FraTrix, which was derived from MaPSaf, was applied over a period of 9 months in the intervention practice. Fratrix didn’t lead to measurable improvements in error managements but lead to better reporting of patient safety incidents.	(EPHPP Statement used for assessment) A strong study which highlighted limitations and implications.
Palacios D [23] 2010	Dimensions of patient safety culture in family practice.	Qualitative case study	Explores the dimensions of patient safety culture related to family practice in UK, USA and Canada.	Global rating of this paper was moderate (Effective Public Health Practice Project)
Incident reporting in primary care setting				
Douglas H [35] 2004	Event reporting to a primary care patient safety reporting system: A report from the ASIPS collaborative.	Incident report analysis	Highest number of events was reported due to communication errors 71% followed by diagnostic and medication errors. A safe reporting system, which relies on voluntary reporting, can be adapted in primary care settings.	All items of STROBE statement covered
Singh R [34] 2006	“Chance favors only the prepared mind”. Preparing minds to systematically reduce hazards in the testing process in primary care.	Prospective study	A proposed approach called as systematic appraisal of risk and its management for error reduction for test process (SARAIMER) was used. Successfully used in medication safety in primary care.	All items of STROBE statement covered
Makeham M [33] 2007	Patient safety events reported in general practice: taxonomy.	Taxonomy	The outline taxonomy of events in general practice provides a complete tool for clinicians describing threats to patient safety and can build an error reporting system.	All items of STROBE statement covered
Marleen S [38] 2010	Patient safety in out-of- hour’s primary care: a review of patient records.	Retrospective	Most frequent incidents occur in out-of- hours primary care were incidents on treatment (56%). Incidents did not result in patient harm. Improved understanding in clinical reason and adherence to guidelines will enhance patient safety.	All items of STROBE statement covered
Zwart D [6] 2011	Central or local incident reporting? A comparative study in Dutch GP out of hour’s services.	Quasi experimental study	Local incident reporting facilitates the willingness to report and faster implementation of improvements. In contrast, central reporting seems better at addressing generic and recurring safety issues. Both approaches should be combined.	All items of STROBE statement covered
Dorien LM Zwart [37] 2011	Feasibility of center-based incident reporting in primary healthcare: The SPIEGEL study	Prospective Observational study	476 incidents reported in 9 months, 62% incidents reported in the reporting week and majority were process oriented. All involved centers initiated improvement strategies due to reported incidents. Locally implemented incident reporting procedure as a tool for managing patient safety is feasible in general practice.	All items of STROBE statement covered
Zwart D [36] 2013	Introducing incident reporting in primary care: a translation from safety science into medical practice	Prospective Observational study	The aim of the study was to understand and describe particular ways primary care physicians make incident reporting procedure part of dealing with safety issues.	All items of STROBE statement covered
Marchon SG [39] 2014	Patient safety in primary health care: a systematic review.	Systematic review	33 articles were selected from 2007 to 2012: 26% on retrospective studies, 44% prospective studies. Frequent method used was incident reporting system 45% and the most relevant contributing factor was communication failure.	8/11 using AMSTAR
Safety climate in primary care setting				
Hoffmann B [35] 2011	The Frankfurt patient safety climate questionnaire for general practice (FraSik): analysis of psychometric properties.	Cross sectional studies	Questionnaire was modified in order to be applicable for general practice. The tool can be used for assessment of the safety climate of general practice.	All items of STROBE statement covered
De Wet C [37] 2012	Measuring perception of safety climate in primary care: a cross- sectional study.	Cross sectional study	Perception of safety climate in the UK primary care with a validated tool specifically designed for it. Measuring safety climate has various benefits at the individual, practice and regional level.	All items of STROBE statement covered
Hoffmann B [36] 2013	Impact of individual and team features of patient safety climate: A survey in family practice.	Cross section studies	FraSik was used to identify potential predictors of the safety climate in family practice in Germany. The overall climate was positive but the health professional’s use of incident reporting and systems approach to errors was fairly rare.	All items of STROBE statement covered
Adverse events in primary care setting				
Sweidan M [41] 2010	Identification of features of electronic prescribing systems to support quality and safety in primary care using a modified Delphi process.	Modified Delphi process.	114 software features were developed which relate to recording and use of patient data, the medication selection process, prescribing decision-making support, monitoring drug therapy and clinical reports. This feature supports safety and quality of prescription of medication in general practice.	Modified Delphi process.
Wong K [40] 2010	A systematic review of medication safety outcomes related to drug interaction software.	Systematic review	No study addressed the benefits and harms or cost effectiveness of drug interactions. The evidence does not support a benefit of software on medication safety or support any practice in this policy.	7/11 using AMSTAR
Singh R [42] 2004	Estimation impacts on safety caused by the introduction of the electronic medical records in primary care.	FMEA	Hazard score was calculated for each error before and 1 year after implementation of electronic medical records. Hazards perceived by staffs decreased in domains of physician –nurses and physicians –chart. But increase in physician- patient and nurse- chart domain.	All items of STROBE statement covered
Joachim S [43] 2011	Effectiveness of a quality improvement program in improving management of primary care practices	Cross sectional study	Primary care practices that completed the European Practice assessments twice over a period of 3 yrs showed overall improvements in practice management, quality and safety and complaint management.	All items of STROBE statement covered

### Safety culture in primary care

Thirteen studies addressed safety culture and tools to assess safety culture in general practice and most (9/13) were cross sectional studies [7, 8, 10, 14–19], the other studies were qualitative interviews [20], a systematic review [21], a retrospective audit [22], randomized control trial [22], mixed methods [23] and a case study [24].

The definition of patient safety culture varied among the articles. A common definition of safety culture was utilized in eight studies, which referred to shared values, perceptions, attitudes, competencies and behaviors within an organization [8, 10, 14, 15, 19–23]. The definition of safety culture was lacking in two articles but they defined patient safety and patient safety incidents respectively [18]. There was one study where patient safety culture was defined as acceptance and actions of patient safety as the first priority in the organization [7] and four articles did not define safety culture [17, 24–26].

Two studies of safety culture utilized a qualitative approach, followed by a survey or an audit. The other eleven studies utililized quantitative tools to assess safety culture. The systematic review included a study by Gaal et al. in the Netherlands that explored the views of primary care doctors and nurses to identify aspects of care linked to patient safety in a qualitative study [16]. Medication safety was most frequently mentioned with incidents occurring in diagnosis and treatment, errors in communication and poor patient doctor relationship were the most common errors in primary care [25]. The aspects that were considered essential for patient safety were; the availability of medical instruments, telephone accessibility and safe electric sockets. General practitioners relied on the skills and knowledge of the practice nurses since most of the patients were seen by them. The GPs did not supervise the practice nurses when providing advice to patients over the phone which they felt was a threat to patient safety. The results of this qualitative study were used to develop a web-based survey, which was one of the first to assess the views of general practitioners (GPs) on patient safety [16] in the Netherlands. They found that GPs were concerned about the maintenance of medical records, prescription and monitoring of medication.

Another Dutch study identified that health care professionals who had a perception and understanding of patient safety had more incidents recorded [26]. All the health professionals surveyed felt that communication breakdown inside and outside the practice was a threat to patient safety and was associated with more incidents [26].

A systematic review on the use of interventions of patient safety that affect safety culture in primary care only included two studies [21]. One of the included studies described the implementation of an electronic medical records system in general practice using the safety attribute questionnaire as a part of patient safety improvements [21]. The authors facilitated two workshops for general practice on risk management and significant audit analysis. The authors concluded that further research was required to assess the effect of interventions on safety culture in primary care [21].

Two main tools were used to measure safety culture; the Manchester Patient Safety Framework (MaPSaF) and the Hospital Survey on Patient Safety Culture (HSOPSC). The Manchester Patient Safety Framework (MaPSaF) [23] was developed to measure the multidimensional and dynamic nature of safety culture and enabled recognition of subcultures within a single organization because subcultures act as a powerful influence on error detection and learning. In addition, the tool provided insights into patient safety culture, facilitated interactive self-reflection about safety culture of an organization, explored differences in perception among different staff categories, helped understand how mature an organization was in terms of safety culture and evaluated interventions which were aimed at improving safety culture. The MaPSaF is founded on Westrum’s typology of organizational communication from 1992, which defined how different types of organizations process information. This typology was expanded upon by Parker and Hudson to describe five levels of progressively maturing organizational safety culture. The MaPSaF measures ten dimensions of safety culture, derived from a literature review on patient safety in primary care and in-depth interviews and focus group discussions with health care professionals and managers. The dimensions are commitment to overall safety, priority given to safety; system errors and individual responsibility; recording incidents and best practice; evaluation incidents and best practice; learning and effecting change; communication about safety issues; staff education and training and team work approach. The tool helped to acknowledge that patient safety was multidimensional and complex, offered insights and demonstrated strengths and weaknesses of a patient safety culture, provided differences in perception among and helped the organization to understand what a mature safety culture in health care might look like. It should not be used to conduct performance management nor to divide or attribute blame when the organization’s safety culture is not sufficiently mature [27]. This tool is best used as a facilitative educational tool for health care providers and managers.

The Manchester Patient Safety Framework (MaPSaF) [14, 22] has been adapted for use in different health systems. The MaPSaF was modified and tested in the New Zealand context to facilitate learning about safety culture and facilitate team communication mentioned in the systematic review [15]. The MaPSaF has been modified for use in the German health system and was renamed the Frankfurt Patient Safety Matrix (FraTix) [22]. This tool was validated and used in a randomized control trial of 60 general practices to determine safety culture at different levels. There were no differences between the general practice physicians’ groups but the intervention group showed improved reporting and management of patient safety incidents than the control group. FraTix appeared to be a good tool for self-assessments aimed at improving safety culture but did not lead to measurable improvements in error management.

The Hospital Survey on Patient Safety Culture (HSOPSC) was developed by the Agency of Health Care and Research for Hospitals in 2004, and has been adapted and modified for other health care settings. It measures healthcare professional’s perspectives towards safety culture at the individual, unit and organizational level. It was pilot tested with more than 1400 hospital employees from 21 hospitals across the USA [28]. The tool was developed after an extensive literature review on safety, accidents, medical errors, safety climate and culture and organizational climate and culture. There were also interviews with hospital staff and surveys. The instrument includes fourteen dimensions, twelve are multiple item dimensions (two safety culture dimensions and two outcome dimensions) and the last two are single item dimensions used to check the validity. This tool has a broad spectrum of applicability has been completed by all types of hospital staff from security guards to nurses, paramedical staff and physicians employed by the organization. In terms of reliability and validity the HSOPSC was found to be “psychometrically sound at the individual, unit and hospital level analysis” [29] in primary care settings. It has since been used in Kuwait, Turkey, the Netherlands and Iran [7, 8, 10, 19]. The dimension most commonly scored among Kuwait, Turkey and Iran was teamwork within the units and the least was non-punitive response to errors. Similarly, the HSOPSC has since been adapted and validated for use in Dutch general practice, and was renamed SCOPE [19], a Dutch abbreviation for systematic culture on patient safety in primary care. Table 2 compares the characteristics of the MaPSaF and HSOPSC.

**Table 2:** Comparision of Manchester Patient Safety Framework (MaPSaF) and Hospital Survey on Patient Safety Culture (HSOPSC)

The Manchester Patient Safety Framework (MaPSaF)	The Hospital Survey on Patient Safety Culture (HSOPSC)
Developed by University of Manchester	Developed by the US agency for Healthcare and Research
Defined patient safety culture according to 10 dimensions: • Continuous improvement • Priority given to staff • System errors and individual responsibility • Recording incidents • Evaluation incidents • Learning and effecting change • Communication personnel management • Staff education • teamwork	Defined patient safety culture according to 12 dimensions: • Frequency of error reporting • Number or error reporting • Supervisors expectations and actions • Organizational learning • Teamwork within units • Communication openness • Feedback and communication about errors\ • Non-punitive response to errors • Staffing • Management support • Teamwork across units • Handoffs and transitions
Reflects on safety culture, highlights differences in perception between staff groups help understand what a mature safety culture might look like and monitor changes over time	The tool can assess safety culture at individual, unit and organizational level.
Deigned to be used in the UK context	Designed to be used globally

Paese [15] used the Safety Attitudes Questionnaire (SAQ) to assess attitudes to safety culture in Brazilian primary care. The survey was conducted among community health agents, nursing technicians and nurses. The SAQ assesses the quality of safety and teamwork standards in a given time in a health care organization. Nine attributes are assessed which are: job satisfaction, teamwork climate, perception of work environment, communication, patient safety, ongoing education, management of the healthcare center, recognition of stress, error prevention by using preventive measures. Patient safety attribute was considered to be an important attribute among the respondents whereas prevention measures to avoid errors were viewed as being a less important attribute.

A case study in a primary care physician practice in the USA explored the impact of a comprehensive risk management program from 2003 to 2009. The program resulted in fewer insurance claims and considerable cost savings thereby enhancing patient safety culture in primary care by implementing risk management program, the program further provided the physicians’ a sense of control over the treatment of malpractice and encouraged them to provide the best care for their patients [24].

### Incident reporting in primary care

Incident reporting to assess patient safety in primary care has grown in importance. There were two types of study under this theme; 1) studies that explored different approaches to incident reporting [6, 30–34] and 2) different mechanism to report incidents [35, 36].

A number of studies have looked at incident reporting mechanisms and no one method was found to be superior. A mixture of methods was required to identify adverse events in primary care. The feasibility of a locally implemented incident reporting procedure (IRP) in primary health centers was evaluated [33]. Introducing IRP in primary care to manage patient safety seemed to be less suitable for dealing with serious adverse events since it neglected the emotional needs of the healthcare workers involved in the medical error [33]. This study further compared the number and the nature of incident reports collected locally (IRP) and from the existing centralized incident reporting procedure. They found that the local incident reporting procedure enabled the health care professionals to control the assessments of their incident reports since the reports remained within the health center. This facilitated organizational learning and in turn increased the willingness to report and facilitated quicker implementation of improvement. The central procedure that collected reports from many settings, appeared to address common and recurrent safety issues more effectively. Therefore, they concluded that both approaches were necessary and should be combined [37].

A systematic review reported on the methodologies to evaluate incidents in primary care, types of incidents, contributing factors and solutions to make a safer primary care. There were 33 included articles and the most universally used method was incident analysis from incident reports (45%). The review did not report on the effectiveness of any specific method for incident reporting nor were specific tools mentioned. The most frequent types of incident were associated with medication and diagnosis errors and the most relevant contributing factor was communication failure among healthcare team [15]. Reviewing medical reports as an approach to incident reporting in primary healthcare was examined in a Dutch study mentioned in the systematic review. This retrospective review identified records with evidence of a potential patient safety incident in out-of-hours primary care and reviewed the type, causes and consequences of the incident. They found that incidents did occur in out-of-hours primary care but that most (70%) did not result in patient harm. The most frequent incident was treatment errors (56%). All incidents were attributed to failures in clinical reasoning because of lack of access to the patient’s medical history, insufficient medical knowledge, high workload, age and being high risk (patients with one or more conditions such as cardiac and vascular disease, asthma/COPD, diabetes, pregnancy, malignancy and immune disease). The mean age for patients with incidents was 52 years compared to 36 years for patients without incidents. Logistic regression analysis identified that the likelihood of an incident increased by 1.03 (95% confidence interval: 1.01 to 1.04) for each year increase in patient age the baseline age used was less or more then 50 [15].

### Safety climate in primary care

Safety climate was assessed in three cross sectional studies using similar definitions of safety climate and safety culture [38–40]. Safety climate was defined as “shared employee perceptions of the priority of safety at their unit and organization at large” [38]. The safety climate was referred to as what was happening in an organization whereas; safety culture explained why it was happening [41].

There was no tool to assess safety climate so Hoffman et al. evaluated the use of the existing Safety Attribute Questionnaire, Ambulatory version which was piloted and modified to be used in general practice. It was renamed the Frankfurt patient safety climate questionnaire for general practice (FraSik) and was used to assess the safety climate in German general practice [38]. FraSik was further assessed in a survey which recongnises strengths and weaknesses of the safety climate of general practice and in addition too, individual and practical features that affect the safety climate perception of health care professionals in primary care [39]. Doctors and health care assistants perceived that safety climate in German general practice was positive and highlighted areas for improvement in patient safety, reporting incidents and cause of errors. A limitation of the study was a low response rate because those that responded to the survey might have an interest in patient safety and therefore more positive response and may not reflect the views other health professionals working in the system [39].

Interestingly, the terms safety climate and safety culture in the studies mentioned above have been used interchangeably although they mean different things. Safety climate is defined as “surface features of the safety culture from attitudes and perceptions of individuals at a given point in time” and “the measurable components of safety culture” [42]. Whereas, a safety culture is the “product of individual and group values, attitudes, competencies and patterns of behavior that determine the commitment to, and the style and proficiency of an organization’s health and safety programs” [14].

### Adverse events in primary care

Two papers reported on adverse events with a focus on medication error [43, 44]. Both the papers related to information technology to improve patient safety and quality of care. A systematic review, which reviewed literature on the use of drug interaction detection software (DIS) [43]. Only four studies met the inclusion criteria and they were not able to address the benefits and harms of drug interaction software for medication safety. There was no published evidence to supports these systems or policies.

An Australian study aimed to identify the features of e-prescribing software that best supported patient safety and quality of care in primary care. A list of 114 features was identified by literature review, key informant and expert groups (Delphi Process). These features could be used to develop software standards by policy makers and could be adapted in other settings and countries, but were not evaluated [44]. Another paper discussed the introduction of an electronic medical record system into primary care because of its impact to improve health care quality. The electronic medical system further includes current practice knowledge, which can support decision making, eventually leading to reduction to practice expenses and further increasing revenues by accurate billing and customer satisfaction [45].

The European Practice Assessment tool was used in a German study to assess the primary care practice focusing on the five domains in primary care practice (infrastructure, people, finance, quality and safety). Two groups where selected, the intervention group is the one which had a previous training in the tool and showed improvement in all the five domains compared to the comparative group which group which didn’t have any previous trainings. This highlighted that there is a benefit to quality improvement when accreditation tools are introduced as a benchmark assessment to improve the health care professional’s performance [46].

## Discussion

Patient safety is critical to health care quality and remains a developmental challenge in primary care in many countries. In addition interventions addressing patient safety culture in primary care are limited compared to secondary care [21].

To improve patient safety, an important first step is to address and understand the safety culture of an organization. Similarly assessment of safety culture helps health care organizations to assess areas for improvement and analyze changes over time [9]. This systematic review has recognized that the most common theme emerging from 2011 onwards was the assessment of safety culture in primary care. An important first strategy to improve all aspects of health care quality is creating a culture of safety within health care organizations [47].

An understanding of the safety culture is vital to improve the problematic practices or attitudes such as miscommunication, adverse events and a non-punitive response to errors, which can lead to an improvement in the safety culture of primary care. Likewise, the measurement of safety culture in primary care can help in the identification of areas for improvement which might cause adverse events and errors. Patient care follow-up, communication openness and work pressure were essential to improve patient safety in primary care [2]. Secondly, another key area for improvement seen in the systematic review was the issue of inadequate numbers of staff and providers to handle patients in primary care, highlighting this as an area that requires attention [7, 8, 10].

Communication breakdown, which affects both safety culture and acts as a contributing factor for incidents, needs to be emphasized and addressed to help strengthen patient safety culture in primary care [19]. Communication openness was seen in the Kuwaiti and Turkey studies as an area of concern [8, 10] unlike in the Iranian and the Dutch studies [7, 19]. The inconsistency between outcomes regarding communication openness might be associated with differences in cultural background where disparagement and disagreement is regarded as blame and thus can lead to loss of occupation or personal relationships among staff and therefore staff tend to avoid it. In general communication openness was found to be a problem in developing and Middle Eastern countries due to the blame culture [9]. Organizations with a positive safety culture constituted a communication policy, established the importance of safety in health care and developed preventive measures.

This systematic review brings to light an emerging literature on patient safety culture in primary care from middle to low income countries. As health care organizations attempt to improve, there is a need to establish a culture of safety an example seen in primary care in Oman.To to achive that, its essential to understand the culture of safety which requires an understanding of the values, beliefs, and norms about what is significant in an organization and what attitudes and behaviors related to patient safety are importand and suitable. Establishing an environment for patient safety may be challenging in Oman because no studies on patient safety have been undertaken in primary care, only hospital care. A further complication is that the health centers are scattered unlike hospitals which is a single unit and in addition the health care workforce includes many nationalities and backgrounds with varying understandings of patient safety from different health care systems.

The insight one may draw from the literature is that, the most reliable and effective strategy for improving the quality of care is in changing the perception of the frontline health care professionals towards patient safety which in-turn will result in reduced adverse events and communication breakdown [47].

The safety of the staff and patients in a health care organization was affected by the extent of safety perceived across the organization. This concept was assessed by two frequently used tools in the systematic review which assessed safety culture in primary care: the Manchester Patient Safety Framework (MaPSAF) and Hospital Survey on Patient Safety Culture (HSOPSC). The HSOPSC tool emerged as the most likely tool to be used in the GCC to assess the safety culture in primary care for the following reasons; firstly, it was used successfully in Kuwait and more recently in Yemen and both countries have a similar GCC primary health systems. Secondly, the same questionnaire has been used to assess the hospital safety culture in other countries in the GCC [48].

Incident reporting is an important aspect for achieving patient safety [6]. There is a need to develop an incident reporting system in primary care in the Middle East within the health centers, similar to hospitals, which is computerized and helps in tracking and following up the incidents. The findings from this systematic review suggest that the system developed should include a local incident reporting system which will record and monitor incidents within the health center along with a centralized reporting system at the ministry of health which can address and monitor incidents which are recurrent and common in primary care [49]. A local approach aids in willingness to report and facilitate quicker implementation whereas a central approach addresses the common and recurrent safety issues [49].

Patient safety in primary care is an emerging field of research in western countries but little has been published from Oman and the other Gulf Cooperation Council Countries (GCC). The Ministry of Health (MOH) in Oman has been working for many years at different levels to improve the quality of health care services and its safety.

Patient safety in primary care can be enhanced in the GCC by introducing 5 yrs plans across primary care. This such example was seen in Oman where they developed a “Vision 2050” which is updated every 5 yrs. Potential areas for improvement are introduced for the next 2020–2025 five-year plan for patient safety in primary care across all the regions of Oman. With the aid of these plans the Ministry of Health, in partnership with the Ministry of Information Technology, are working together to achieve information transfer, linkage of patient information between health centers, secondary care and hospitals so that the civil identification number can be used as a single identification number to access all patient health information across the health institutions.

## Conclusion

This systematic review reveals that the most important first step is the assessment of safety culture in primary care which will provide basic understanding to safetyrelated perceptions of the health care providers. The most commonly used safety culture assessment tool is the HSOPSC which aids in identifying areas for improvement at the individual, unit and organizational level. This review recognized that safety culture in primary care should be assessed on a regular basis to evaluate the effectiveness of safety in health institutions.

Furthermore, results from this review will be used to inform an empirical study of safety culture in primary care in Oman using the Hospital Survey on Patient Safety Culture (HSOPSC) tool, with a view to developing a template for the development of safety culture in primary care in the context of rapid economic growth.

## Additional file

Additional file 1: PRISMA flowchart. The completed PRISMA flowchart for the systematic review. (DOC 57 kb)

## Abbreviations

AMSTAR: Assessing Methodological Quality of Systematic Review
EPHPP: Quantitative studies were assessed by Effective Public Health Practice Project
GCC: Gulf Cooperation Council
HSOPSC: Hospital survey on patient safety culture
IOM: International Institute of Medicine
MOH: Ministry of Health
PRISMA: Preferred Reporting Items for Systematic Reviews and Meta-Analyses
SAQ: Safety attribute questionnaire
STROBE: Cross sectional studies were evaluated by using Strengthening the Reporting of Observational studies in Epidemiology
WHO: World Health Organization
