# Supplementary material for: Patient safety and safety culture in primary health care: a systematic review
Source: BMC Fam Pract. 2018 Jun 30;19:104. doi: 10.1186/s12875-018-0793-7 (PMC6026504; doi:10.1186/s12875-018-0793-7)
Supplement: Supplementary file 1 — PRISMA flowchart. The completed PRISMA flowchart for the systematic review. (DOC 57 kb) [file 12875_2018_793_MOESM1_ESM.doc]

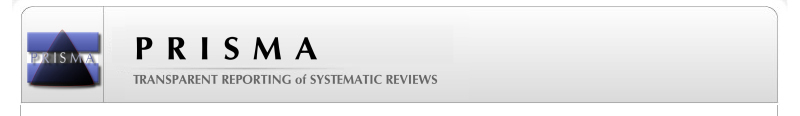
**PRISMA 2009 Flow Diagram**

**Screening**

**Included**

**Eligibility**

**Identification**

Records identified through database searching
(n =3268)

Records after duplicates removed
(n = 3072)

Records screened
(n = 261)

Records excluded
(n = 2811 )

Studies with outcome which fulfilled the inclucion criteria

(n=61)
(n = )

Narrative reviews, full text studies not in English, studies focusing on single service in primary care excluded

(n=33)

Studies included in quantitative synthesis (meta-analysis)
(n = 28)

No abstract (n=83)

No intervention (n=117)
